# Psychogenic Non-epileptic Seizures and Pseudo-Refractory Epilepsy, a Management Challenge

**DOI:** 10.3389/fneur.2020.00461

**Published:** 2020-06-02

**Authors:** Francesca Anzellotti, Fedele Dono, Giacomo Evangelista, Martina Di Pietro, Claudia Carrarini, Mirella Russo, Camilla Ferrante, Stefano L. Sensi, Marco Onofrj

**Affiliations:** ^1^Department of Neurology, “SS Annunziata” Hospital, Chieti, Italy; ^2^Department of Neuroscience, Imaging and Clinical Science, “G. D'Annunzio” University of Chieti-Pescara, Chieti, Italy; ^3^Behavioral Neurology and Molecular Neurology Units, Center for Advanced Studies and Technology (CAST), University G. d'Annunzio of Chieti-Pescara, Chieti, Italy; ^4^Institute for Mind Impairments and Neurological Disorders, University of California, Irvine, Irvine, CA, United States

**Keywords:** PNES, functional neurological disorder, pseudo-refractory epilepsy, dual diagnosis, PNES psychopathology, PNES Imaging, PNES treatment

## Abstract

Psychogenic nonepileptic seizures (PNES) are neurobehavioral conditions positioned in a gray zone, not infrequently a no-man land, that lies in the intersection between Neurology and Psychiatry. According to the DSM 5, PNES are a subgroup of conversion disorders (CD), while the ICD 10 classifies PNES as dissociative disorders. The incidence of PNES is estimated to be in the range of 1.4–4.9/100,000/year, and the prevalence range is between 2 and 33 per 100,000. The International League Against Epilepsy (ILAE) has identified PNES as one of the 10 most critical neuropsychiatric conditions associated with epilepsy. Comorbidity between epilepsy and PNES, a condition leading to “dual diagnosis,” is a serious diagnostic and therapeutic challenge for clinicians. The lack of prompt identification of PNES in epileptic patients can lead to potentially harmful increases in the dosage of anti-seizure drugs (ASD) as well as erroneous diagnoses of refractory epilepsy. Hence, pseudo-refractory epilepsy is the other critical side of the PNES coin as one out of four to five patients admitted to video-EEG monitoring units with a diagnosis of pharmaco-resistant epilepsy is later found to suffer from non-epileptic events. The majority of these events are of psychogenic origin. Thus, the diagnostic differentiation between pseudo and true refractory epilepsy is essential to prevent actions that lead to unnecessary treatments and ASD-related side effects as well as produce a negative impact on the patient's quality of life. In this article, we review and discuss recent evidence related to the neurobiology of PNES. We also provide an overview of the classifications and diagnostic steps that are employed in PNES management and dwell on the concept of pseudo-resistant epilepsy.

## Introduction

Psychogenic non-epileptic seizures (PNES) are relatively common disorders managed by epilepsy centers (1) and consist of paroxysmal motor, non-motor, or behavioral alterations that resemble epileptic seizures without EEG correlates. These disorders are considered to reflect the response to distress or behavioral problems (2). According to the DSM 5, PNES are a subgroup of conversion disorders (CD) or, as indicated by the ICD 10, a dissociative disorder (3). Patients with PNES exhibit a high percentage of psychiatric comorbidities like personality and post-traumatic stress disorders, anxiety, and major depressive disorders (4). A childhood history of abuse, psychiatric comorbidities, and the female gender are all risk factors for CD (5). Trauma, brain injury, surgical procedures (6), or learning disability (7) have also been considered to facilitate the ensuing of PNES. For a long time, PNES have been considered disorders generated in the absence of biological and organic substrates. Thus, most of the attention has been focused on the psychosocial correlates of the condition (8, 9) and PNES patients have been mainly investigated and treated with psychoanalytic/psychodynamic approaches. The psychosocial origins of PNES have been largely endorsed by specialists as well as patients who often find it difficult to reconcile themselves with the idea of suffering from a disorder that lacks an organic basis (10, 11). However, over the last two decades, the use of neuroimaging techniques and functional connectivity studies have provided evidence to further understanding of the neurobiological underpinnings of this condition (12–15).

The current systematization favors the notion that PNES results from the convergence of genetic, neural, and environmental factors that synergistically act in the context of permissible psychological conditions/disorders (16). The management of PNES patients is different compared to what employed for epileptic patients, and accurate diagnosis of PNES is essential. The lack of prompt identification of PNES in epileptic patients can lead to potentially harmful increases in the dosage of anti-seizure drugs (ASD) as well as erroneous diagnoses of drug-resistant epilepsy. Epilepsy and PNES can coexist in a “dual diagnosis” condition. This condition mandates accurate discrimination between real epileptic seizures from PNES as the lack of pharmacological response to ASD of PNES events may lead to a diagnosis of a (pseudo)pharmacoresistant epilepsy.

In this article, we review and discuss recent evidence related to the neurobiology of PNES; we provide an overview of classifications and diagnostic steps that are employed in PNES management. Finally, we stress the concept of “pseudo-refractory epilepsy” which represents a central issue in the treatment and management of epileptic patients who are also presenting PNES.

## Epidemiology of PNES

PNES are relatively common disorders that are managed by neurologists, particularly in epilepsy clinics. The incidence of PNES is estimated to be in the range of 1.4–4.9/100,000/year, and the prevalence range is between 2 and 33 per 100,000 (2, 17). Five to 10% of the outpatients of epilepsy clinics and 20–40% of the inpatients of epilepsy monitoring units exhibit PNES. PNES usually begin young adulthood, although the disorder can occur at any age (18–20). A confirmed diagnosis is often significantly delayed, thereby leading patients to receive unnecessary treatments for years. Neurobiological, social, and vulnerability factors may explain why PNES are predominantly seen in females (16). Intriguingly, the prevalence of epilepsy in patients with PNES has been estimated to vary in a wide range from 5.3 to 73% (21). Although previous studies did not report the exact figure of this condition, a recent review has shown a prevalence of epilepsy among PNES patients around 22%, whereas the prevalence of PNES among epilepsy patients is 12% (22). This higher incidence has brought specialists to speculating that epilepsy may be a contributing risk factor for developing PNES not only because of predisposing biological mechanisms but also because, in subjects affected by genuine epilepsy, the experience of epileptic seizures may provide an opportunity for model learning (11, 23).

## PNES Classifications

A practical semiological classification of PNES must address proper diagnosis, the etiological systematization as well as help the management of patients. Experts have provided several classification systems based one the age, semiology, or video-EEG analysis (19, 24–26). At the beginning of the century, Gröppel and colleagues (27), taking into account the semiology of the disorder, classified PNES in: (1) “Major motor,” a form characterized by the association of clonic and exaggerated motor movements of the upper and/or lower extremities, pelvic thrusting, head movements, and tonic posturing of the head; (2) “Minor motor or trembling,” a form characterized by trembling of the upper and/or lower extremities; and (3) “Atonic psychogenic seizures,” a form characterized by falls as the only symptoms. In the same years, Selwa and collaborators (28) proposed six types: (1) “Catatonic PNES”; (2) “Trashing PNES”; (3) “Automatisms”; (4) “Tremor”; (5) “Intermittent PNES”; and (6) “Subjective PNES”. Later on, Seneviratne and colleagues (29) offered a new classification structured in six categories: (1) “Rhythmic motor PNES”; (2) “Hypermotor PNES”; (3) “Complex motor PNES”; (4) “Dialeptic PNES”; (5) “Non-epileptic auras” or (6) “Mixed PNES”. Hubsch and colleagues (26) have then proposed a more detailed cluster analysis that identified five subtypes, based on the clinical features of the attacks, as (1) “Dystonic attack with primitive gestural activity”; (2) “Paucikinetic attack” with preserved responsiveness; (3) “Pseudosyncope”; (4) “Hyperkinetic prolonged attack with hyperventilation and auras” or (5) “Axial dystonic prolonged attack”. Dhiman and colleagues (19) have recently modified a previous classification employed in children with PNES, and proposed five subtypes: (1) “Abnormal motor” (hypermotor movement of the whole body or only of the head and neck); (2) “Affective/emotional behavior phenomena”; (3) “Dialeptic Coma-like state”; (4) “Aura”; or (5) “Mixed”.

All these past classifications shared a complex structured organization based on an accurate clinical video-EEG description of PNES. According to some studies (28, 30), outcome of PNES may vary among different clinical types, and it was also believed that different psychopathologic aspects underpinned all these manifestations. In fact, psychologists and psychiatrists documented a variety of different personality profiles and psychological etiologies including conversion disorders, depression, post-traumatic stress disorder, anxiety, emotional trauma, dissociative disorders, psychosis, and impulse control problems have been implicated in the pathogenesis of different clinical types of PNES. However, complex classifications encounter some limits in the daily clinical routine application especially if they are far different from the classification of true seizures.

Finally, in 2016, Magaudda et al. (25) proposed a classification based on the notion that all the PNES subtypes are similar to the subtypes of true seizure, and have, therefore, offered four categories corresponding to the ones most frequently found in their clinical experience as (1) “Hypermotor”; (2) “Akinetic”; (3) “Focal motor”; or (4) PNES with “Subjective symptoms”. This latest classification was considered useful and practical, providing a good classification tool that can allow standardization across future studies (24).

A synopsis of all the classifications is provided in Table 1. Of note, it is indisputable that in the end all these classifications can be simply reconfigured in terms of “motor” vs. “non-motor” PNESor PNES with or without “unresponsiveness”.

**Table 1:** PNES classifications.

Gröppel et al. (27)	1. Major motor 2. Minor motor or trembling 3. Atonic psychogenic seizures
Selwa et al. (31)	1. Catatonic 2. Trashing 3. Automatisms 4. Tremor 5. Intermittent 6. Subjective
Seneviratne et al. (29)	1. Rhytmic motor 2. Hypermotor 3. Complex motor 4. Dialeptic 5. Non-epileptic auras 6. Mixed
Hubsh et al. (26)	1. Dystonic attack with primitive gestural activity 2. Paucikinetic attack (with preserved responsiveness) 3. Pseudosyncope 4. Hyperkinetic prolonged attack with hyperventilation and auras 5. Axial dystonic prolonged attack
Dhiman et al. (19)	1. Abnormal motor 2. Affective emotional behavior phenomena 3. Dialeptic coma-like state 4. Aura 5. Mixed
Magaudda et al. (25)	1. Hypermotor 2. Akinetic 3. Focal motor 4. PNES with “subjective symptoms”

## PNES Pathophysiology

### Psychopathology Aspects

As PNES is a group of symptoms and not a disease or a syndrome, the underlying etiology is expected to be heterogeneous. The psychopathologic aspects of PNES and other conversion disorders (CD) have been documented for centuries and summarized in a statement by Stone and colleagues (32) as “patients who show difficulty in expressing conflicts verbally, sometimes express distress somatically.” Despite the presence of a wealth of studies that have described the functional and structural neuroimaging correlates as well as the serologic, cardiac, and electrophysiological features occurring in patients affected by functional neurologic disorders and CD (33), a unifying neurophysiological model for these conditions is still missing. *Dissociation* is considered by many specialists a key mechanism of the disorder, and people who experience PNES often exhibit a variety of dissociative symptoms (34). According to the DSM-5, dissociation is defined as “a disruption and/or discontinuity in the normal integration of consciousness, memory, identity, emotion, perception, body representation, motor control, and behavior” (35). Dissociation is considered a defense mechanism that helps the individual in coping with traumatizing events. In that sense, PNES often follow stressful or traumatic events that are generating a dissociation of the mental organization (36).

**Figure 1:**
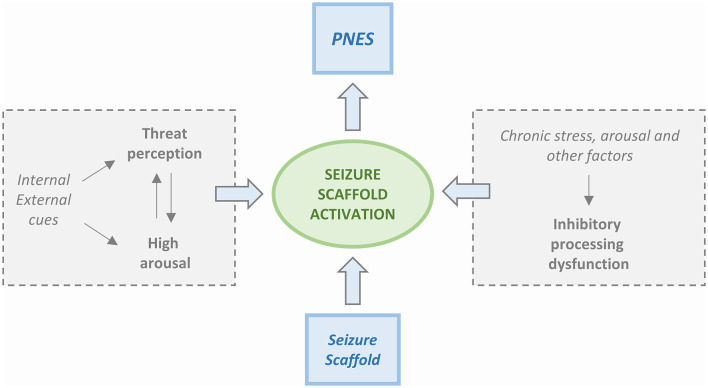
Simplified scheme of the Integrated Cognitive Model (ICM). PNES result from the automatic execution of acquired mental representations of seizures (i.e., the enacting of a “*seizure scaffold”*). The seizure scaffold consists of a sequence of perceptions and motor activities shaped by experiences such as inherent reflexes (i.e., freezing movements, startle, wandering) or physical symptoms (i.e., of pre-syncope, dissociation, hyperventilation, head injury). Seizure scaffolds can be triggered by a range of internal or external stimuli. The process often occurs in response to increases in autonomic arousal. However, the seizure scaffold is more likely to be triggered in the presence of dysfunctional inhibition that can be due to chronic stress but also driven by “physical” causes like concurrent illness, effects of medication, etc. Patients usually experience the enactment of the seizure scaffold as non-volitional, although they may be able to inhibit it voluntarily.

Four PNES etiological models are available. The first model is based on the Freudian construct (37) and posits that PNES is a physical manifestation of emotional stress. The second model, proposed by Moore and Baker (38), had suggested that PNES results from learned behavior and operant conditioning. Two more recent models have been centered on the presence of dissociative mechanisms. Bowman (39) has proposed that PNES results from dissociated memories or mental functions that are set in motion by traumatic events. Baslet (40) has instead proposed that PNES is an acute dissociative response to a threat or a state of high arousal. We have not achieved a “*one size fits all”* model, and, realistically, each one of the four can only partly explain the underlying mechanisms of PNES. However, the new integrated cognitive model (ICM) put forward by Brown and Reuber (3) appears to be a step in the right direction toward the identification of a unitary explanation. According to the model (Figure 1), PNES results from the consequences produced by altered stimuli on the activation of memory networks. The model is based on the alteration of physiological functioning in which the response to a stimulus depends on the familiarity with it. Accordingly, a familiar stimulus, already represented and stored in memory networks, generates an automated execution of a motor program (41) while if the stimulus is unfamiliar and memory networks have not been primed, a non-automated response is generated. The physiological model takes into consideration also the activation of secondary attention systems that are in charge of the “go” for responses to be executed. Action is, therefore, perceived as voluntary and self-controlled.

Upon PNES, an altered pattern of automatic responses that do not match or are rooted in reality is generated. PNES are, therefore, caused by a “rogue representation,” a distorted perception of a prior or unfamiliar stimulus. At difference with physiological functioning, the automatic response is experienced as involuntary and unwanted. According to the model, PNES production is influenced by the patient background of life experiences that include memories of seizures (experienced or witnessed) as well as by an intrinsic repertoire of automatic responses to emotions like anger, fear, or disgust. In summary, while in healthy people, automated behavior, even when stereotypical, is not elicited by emotional triggers, PNES patients exhibit an abnormal coupling between emotional triggers and the production of automatic behavioral responses that take the form of pseudo-seizure attacks. This sequence of perceptions and actions is relatively stable but not completely uniform. As such, the pathophysiological setting has much in common with the essential constituents of classical conditioning. Furthermore, these patients are unaware of the connection between the emotional state that has acted as a trigger and the resulting dysfunctional automatic behavior. Table 2 summarizes the main psychopathologic theories of PNES.

**Table 2:** PNES models.

*Freudian model*	PNES is a physical manifestation of emotional stress
*Moore and Baker model*	PNES results from learned behavior and activated via operant conditioning
Dissociative models by *Bowman and Baslet*	PNES results from dissociated memories or mental functions that are set in motion by a traumatic event (Bowman)
	PNES is an acute dissociative response to a threat or a state of high arousal (Baslet)
Integrated Cognitive Model (ICM) by *Brown and Rewber*	PNES results from an altered stimulus that in physiological conditions would have caused the activation of memory networks; the response to the stimulus depends on the familiarity with it. A familiar stimulus, already represented and stored in the networks, generates the automated execution of a motor program. If the stimulus is unfamiliar and memory networks are not primed, no-automated responses are generated. A secondary attention system that selects responses to be executed is also involved. Action is perceived as voluntary and self-controlled.

### Neurobiology of PNES

A wealth of studies has provided insights into the psychosocial features of PNES (8, 41), but the biological underpinnings of the disorder have received much less attention and are still poorly understood. However, an emerging and growing body of evidence has finally started to unravel the neurobiological basis of PNES (42–44). Structural and functional connectivity studies (42) have shown that PNES patients exhibit network instability and distinct alterations of functional connectivity patterns (12, 43, 45–52). This evidence provides the missing neurophysiological correlates of dissociative mechanisms that let emotions to influence executive control and produce symptoms. However, it is still unclear whether these findings are specific to PNES or are instead tied to other comorbidities like depression, traumatic brain injury, etc., conditions that, it should be underlined, are frequently found in PNES patients (11, 12). Functional connectivity studies investigating CD patients have found distinct connectivity patterns that link the emotional and executive systems (53). These findings indicate that PNES patients exhibit a distinct activation of patterns of functional connectivity that occurs between the insula and the parietal associative areas that are involved in motor planning. These data support the presence of functional connections between regions that control emotional processing and areas in charge of motor planning, a process that occurs while bypassing the conscious motor control (54). This hypothesis is in support of the ICM model and the one proposed by Baslet (40) that postulates that PNES can be interpreted as the paroxymal occurrence of episodes of dysfunctional behavior that are facilitated by the presence of unstable cognitive-emotional-attention systems.

#### Morphological Brain Changes in Patients With PNES

To date, only two morphological studies have examined the structural changes occurring in the brain of individuals with PNES. One study by Labate et al. (46) indicated that PNES patients, when compared to healthy controls, show significant gray matter volume reductions in the cerebellum, the right precentral and middle frontal gyrus, the right anterior cingulate cortex, and the right supplementary motor area as well as signs of cortical thinning in the right precentral gyrus, the right superior frontal gyrus, the right precuneus, and the right paracentral gyrus. A second, surface-based morphometric study by Ristić et al. (47), differed somewhat from the findings reported by Labate et al. (46) and indicated that, compared to healthy subjects, PNES patients exhibit increased cortical thickness in the left insula, the left and right medial orbitofrontal, and left orbitofrontal regions, as well as the decreased cortical thickness of the right precentral gyrus, the right entorhinal, the right lateral occipital, and left precentral areas. Both studies revealed the presence of decreased regional cortical thickness in PNES patients; however, the study by Labate et al. (46) indicates decreases that occur only in the right hemisphere while the study by Ristić et al. (47), has shown bilateral decreases as well as increases in limbic and orbitofrontal regions (47). It should be pointed out that morphometric changes may also occur for non-pathological reasons (55).

#### Structural and Functional Connectivity Patterns in PNES Patients

Another way to look at the structural brain changes that more closely match brain functioning is through the investigation of the strength and integrity of the connectivity that spans across distinct brain regions. A study (48), had employed diffusion tensor imaging (DTI) indices to examine the white matter based structural connectivity of the uncinate fasciculus of PNES patients. The uncinate fasciculus is a critical tract for the connection of the medial prefrontal regions with limbic areas that play an essential role in the production and modulation of emotion and memory processes. The study revealed the presence of lateralization of the connectivity of the uncinate fasciculus. In PNES patients, the authors found significantly higher numbers of streamlines (the visual and statistical DTI-based representations of white matter tracts) in the right uncinate fasciculus, a lateralization that is not present in healthy controls. This connectivity pattern suggests that individuals with PNES exhibit preferential and stronger connections between the prefrontal and limbic regions in the right hemisphere. The study also suggested that the right lateralization has detrimental effects on emotion regulation. However, another DTI-based study (49) found the presence of increased connectivity only in the left uncinate fasciculus and superior temporal gyrus. The study also reported increased connectivity in the corona radiata, and internal and external capsules, areas that are critically associated with motor functions. Thus, DTI-related data are, to date, somewhat contradictory. Stronger structural connectivity between the prefrontal and limbic regions may predispose to PNES by favoring emotion dysregulation; however, it is not clear whether the enhanced connectivity of the uncinate fasciculus potentiates the ability to downregulate emotional responses rather than cause emotion dysregulations. Furthermore, given the intrinsic complexity of the structural connectivity of the white matter and the large number of subcortical connections, it is reasonable to consider that other tracts are involved in the process.

The use of fMRI offers additional evidence for the brain-related features of PNES. To date, only one study, employing DTI as well as resting-state fMRI (rs-fMRI), had simultaneously evaluated the structural and functional connectivity features exhibited by PNES patients (42). The study found that PNES patients exhibit significantly decreased strength of structural and functional connectivity occurring in brain regions that are involved in attention and sensorimotor processing as well as areas that are part of the Default Mode Network (DMN). A follow-up study (43), employing functional connectivity density mapping based on the same rs-fMRI data, found that PNES patients show bilateral differences in the long-range and short-range functional connectivity that involves the frontal, sensorimotor, cingulate, insular, and occipital regions. A study (56), focused on the distinct functional connectivity patterns of the insula and comparing PNES patients with healthy controls has shown that functional connectivity maps relative to the left ventral anterior insula, the right dorsal anterior insula, and the right posterior insula exhibit significant differences in connectivity values in the patient group. A follow-up rs-fMRI study by the same group (50) re-analyzed the dataset and found that, compared to healthy controls, PNES patients show increased synchronous activity mainly occurring in the dorsolateral prefrontal cortex, parietal, and motor regions. PNES patients also show decreased activity in the right triangular inferior frontal gyrus, an area that is part of the ventrolateral prefrontal cortex and associated with the modulation of response inhibition (50). These findings suggest that alterations of the functional connectivity of brain regions associated with attention, memory, emotion processing, sensory, and motor functions are compromised in PNES patients. These alterations, likely resulting from life experiences, generate aberrant sensorimotor interactions that escape the conscious control of the individual. Moreover, it can be hypothesized that the inability to inhibit behavioral outputs in response to emotional stimuli (50) results from the dysfunctional hyper-connectivity that occurs between subregions of the insula and selected sensorimotor, parietal, and occipital regions (56). The process can be at the basis of maladaptive long-term enhanced vigilance to external stimuli (43). In summary, these findings provide support to the idea that PNES is produced by alterations in cognitive-emotional-behavioral mechanisms that result from adverse life experiences and/or maladaptive experiential learning (3, 50, 57).

#### Positron Emission Tomography (PET) Findings in PNES Patients

Fluorodeoxyglucose (FDG) Positron-Emission Tomography (PET)-based evidence indicates that compared to healthy subjects, PNES patients exhibit significant hypometabolism in the right inferior parietal/central brain regions as well as, bilaterally, in the anterior cingulate (44). These findings provide support for two pathophysiological mechanisms involved in PNES: the emotion dysregulation that involves the anterior cingulate hypometabolism and dysfunctional processes associated with self-awareness/consciousness of oneself and the environment that are associated with the hypometabolism of the right inferior parietal cortex. Although intriguing, this study has significant limitations related to the employed exclusion criteria set to exclude co-existing psychopathologies in the recruited patients, a key confounding factor especially when considering the role of the anterior cingulate cortex in the production of anxiety and post-traumatic stress disorders (PTSD) (58, 59).

#### Single-Photon Emission Computed Tomography (SPECT) Findings in PNES Patients

Epileptic patients, evaluated with SPECT scans during ictal events, show hyperperfusion of the epileptic focus while, in the interictal period, the region is hypoperfused (60). Thus, computerized quantifications of the ictal, inter-ictal, and postictal changes in regional cerebral blood flow may be useful to differentiate epileptic from non-epileptic episodes (61–63). Some studies have indicated the possibility of abnormal SPECT findings in the post-ictal phase exhibited by PNES patients (61, 63). A note of caution is required, as most authors concur in the conclusion that solid SPECT-based evidence is still missing in PNES patients. It should also be underlined that these findings are difficult to interpret, given the small sample size and the presence of psychiatric comorbidity in most of the investigated PNES patients (64).

#### Genetic and Other Intrinsic Factors

Genetics of PNES is growing. However, the identification of specific mutations is still missing. Some evidence can be inferred by the analysis of single nucleotide polymorphisms that have been associated with a range of psychiatric disorders (i.e., autism spectrum disorders, attention deficit hyperactivity disorder, bipolar disorder, major depressive disorder, schizophrenia). These studies have provided the first genome-wide based evidence that many distinct psychiatric disorders share individual and aggregate genetic risk factors. To date, the only acknowledged genetic risk factor for the development of PNES is gender (11, 65). The evaluation of this risk factor goes along with the growing field of gender-based neurology, as recent evidence indicates the presence of distinct sex-dependent differences that shape the functional connectivity of regions involved in emotional and cognitive processing (66).

## Diagnosis

### The Importance of an Early Diagnosis

About one-quarter of patients who are sent for video-EEG monitoring in cases of suspected pharmaco-resistant epilepsy are then found to suffer from PNES (21). PNES patients commonly experience delays in the diagnosis and/or receive inappropriate treatment. Physicians often fail to communicate and explain the condition to patients. As ASD are of no use in PNES and may exacerbate the disorder (67), early and accurate diagnosis, as well as the exclusion of epileptic seizures and other paroxysmal disorders, is of paramount importance. According to the National Association of Epilepsy Centers (NAEC) Guidelines (68), if PNES are suspected, prompt referral to an epilepsy center is required as early diagnosis of PNES is associated with better outcomes. Recent evidence shows that delays in PNES diagnosis are common. Some factors may contribute to the delay and include demographic (i.e., young age), clinical variables (i.e., the association of PNES with trauma and body injury, or physician-related variables (i.e., ASD history).

### Differential Diagnosis Between PNES and Epileptic Seizures Based on Clinical Features

Clinical and semiology information can help in distinguishing PNES from epileptic seizures. Avbersek and Sisodiya (69), for instance, state that the criterion: “occurrence from sleep” has a 100% specificity for epileptic seizures. Unfortunately, approximately half of the PNES patients has a positive history of ictal events occurring “upon arising from sleep” (70), thereby indicating that the sleep-related criterium cannot be taken as good evidence for epilepsy unless the events occur only upon sleep (70). The reduced semiotic congruency of PNES episodes, when compared to genuine seizures, is another criterium that has been employed to differentiate the two disorders, but also a matter of controversy among experts (71). A recent retrospective semiotic study concluded that neither the stereotypic quality of the ictal episodes nor the variability of clinical presentations should be used as a valid criterion to differentiate PNES from epilepsy (28). Another discriminating criterion concerns the length of ictal events. As a rule of thumb, it is assumed that episode duration in PNES is longer than what occurring in genuine seizures (31). Real seizures exhibit a well-characterized onset, reach the peak of the clinical manifestations within 70 s after the onset (72), and are followed, within a few minutes, by the ensuing of the ictal offset. In the case of tonic-clonic seizures, motor manifestations that last longer than 2 min strongly indicate the need for differential diagnosis with PNES (31). A ictal episode lasting more than 10 min is most likely due to PNES (69). Epilepsy and PNES can be differentiated by a broad array of distinct symptoms and signs. It is true that PNES patients commonly exhibit asynchronous limb movements, out-of-phase clonic activity, rhythmic shaking movements with episodes of inactivity, side-to-side head movements, pelvic movements, dystonic body posturing, closure of the eyes during the event as well as enacting of non-stereotypical seizure patterns. However, it should be remembered that none of these signs are pathognomonic for PNES. In the case of generalized tonic-clonic seizures (GTCS), the differential diagnosis is eased by the fact that a genuine GTCS evolves through a stereotyped, structured progression, typically beginning with an ictal vocalization, followed by the bilateral adduction and external rotations of the limbs, the tonic extension of all four limbs, and then the production of diffuse clonic jerking movements before the ictal offset. By contrast, patients with PNES exhibit vocalization not only at the beginning of the event but also throughout the whole ictal episode. The vocalization can fluctuate, persist, and be present, with different pitch intensities, throughout the whole course of the “ictal” episode. Moreover, movements produced in hypermotor PNES are usually showing less organized spatial patterns and characterized by movements of variable rhythmicity and amplitude.

Focal-to-bilateral tonic-clonic seizure can be frequently preceded by a focal seizure described as “epileptic aura” according to the past nomenclature. Auras are also common in PNES and cannot be considered a hallmark of epilepsy. In a recent study (73), the authors investigated the incidence of aura in patients with PNES, a clinical sign often present in these patients. Unfortunately, PNES auras are not different from the ones exhibited by epileptic patients.

Subjective symptoms, urinary incontinence, at night, and ictal self-injury are often associated with genuine seizures; however, again, none of these signs is pathognomonic for epilepsy as more than one-third of the PNES patients reported the same symptoms (74). Thus, these symptoms cannot be used as different and discriminating features of the two conditions.

Table 3 depicts the distinct clinical features of PNES and epilepsy that can help to discriminate the two conditions.

**Table 3:** Differential diagnostic features PNES and epileptic seizures.

	**PNES**	**Epileptic seizures**
Aura	Less frequent	More frequent
Length of ictal events	>10 min	<70 s (<2 min for tonic-clonic seizures)
Seizure patterns	Non-stereotypical, less organized spatial patterns, variable rhythmicity, and amplitude of movements	Stereotypical and organized progression
Clinical findings	Asynchronous limb movements, out-of-phase clonic activity, rhythmic shaking movements with episodes of inactivity, side-to-side head movements, pelvic movements, dystonic body posturing, closure of the eyes during the event	Bilateral adduction and external rotations of limbs followed by tonic extension of all four limbs, then the production of diffuse clonic jerking movements before the ictal offset
Vocalization	Present not only at the beginning of the event, can fluctuate, persist and be present, with different pitch intensities, throughout the whole course of the ictal episode	At the beginning of the seizures
Subjective symptoms	Less frequent	More frequent
Urinary incontinence	Less frequent	More frequent
Occurrence at night	Less frequent	More frequent
Ictal self-injury	Less frequent	More frequent

*A word of caution is required as, according to recent evidence, the prevalence of aura, subjective symptoms, urinary incontinence, night occurrence of ictal events, and self-injury in PNES patients is higher than what previously researches reported, thereby making challenging to discriminate PNES and epileptic seizures only based on clinical signs*.

### Video-EEG Monitoring: The Diagnostic Gold Standard

Prolonged video-EEG monitoring with ictal recording is considered the optimal test for the diagnostic ascertainment of PNES. However, unfortunately, some types of seizures either do not exhibit ictal EEG abnormalities, or EEG changes are concealed by the movements (i.e., frontal lobe seizures), thereby making the clinical differentiation with PNES difficult. The diagnosis of PNES should, therefore, take into account a combination of data. To that aim, the combination of the patient history, witness reports, clinician observations, ictal and interictal EEG as well as ictal video-EEG can be used (70, 75). Nowadays, home video recording is available and significantly helps the diagnostic process. While accurate evaluation of clonic movements, tremors, or thrashing movements is difficult when assessed only on what referred by eyewitnesses, the examination of video recording by expert clinicians significantly helps in producing a correct differential diagnosis (31). One caveat on the use of home-based EEG recording concerns the fact that they rarely capture the beginning of the ictal event. It is also important to note that the postictal phase of some epileptic seizures may look like PNES.

In summary, an accurate diagnosis is produced when (1) the patient history is compatible with PNES; (2) the semiology is coherent with the distinct features of PNES, as assessed by an expert clinician employing video-EEG monitoring; and (3) the episode unmistakably lacks epileptiform activity in all the phases (i.e., immediately before, during or after the ictal event). A useful rule of thumb to suspect PNES is “the rule of 2 s” that indicates that likely PNES patients, subjects exhibiting at least two normal interictal EEG along with at least two episodes per week and resistance to two antiepileptic drugs. The rule yields an 85% positive predictive value for PNES (76).

Also, seizure-related induction procedures exhibit good sensitivity and excellent specificity for PNES and help to shorten the length of the hospitalization time required for the diagnosis (77). These techniques are not universally accepted in clinical practice and currently employed by about 39–73% of the US epilepsy centers (78). A critical issue concerns the fact that there are no standardized induction techniques. The induction encompasses an array of triggers that range from simple verbal suggestions to the employment of placebos like saline injections, perfumes and olfactive stimulants, sham application of EEG-electrodes, the use a soaked pad on the patient's neck or a vibrating tuning fork on the forehead as well as the use of standard activation procedures employed in EEG like hyperventilation and photic stimulations. The use of saline injections has, for instance, a diagnostic sensitivity in the range of 60 to 90% (79). While clinically useful, the employment of induction procedures raises ethical concerns and is a matter of debate (80–88). A major ethical issue is posed by the levels of deception involved in the information provided to patients. In that regard, communication strategies have been commonly divided into three categories: (1) “explicitly deceptive,” a situation in which an untruthful statement is madelike when a patient is told that “*a seizure will be produced […] by placing a patch on the arm*”; (2) “truthful but omissive”when the information is technically truthful and the patient, for example, is told “*we will inject an IV drug that will perhaps help in inducing the usual spell”* but the words “epileptic seizure” are omitted to avoid lying to the patient (89); or (3) “explicitly open” when the provided information is technically correct, the psychological origin of the condition is introduced as a possibility before the induction, and the patient is made aware of the possible occurrence of both epileptic and psychogenic seizures (like during hyperventilation and photic stimulations). A recent review by Stoyan Popkirov and colleagues (89) has analyzed changes in communication methods over the years and indicates a predominant tendency toward the use of more honest strategies. Some of the ethical concerns can be circumvented by using only activation procedures that are routinely employed in EEG. Hyperventilation and photic stimulation, in fact, exhibit a diagnostic power comparable with the induction with placebo (83).

The PNES diagnosis is possible, probable, clinically established, or documented:

– **Possible PNES**: cases in which a witness or the patient reports ictal events, and the interictal EEG is normal. An abnormal interictal EEG can also be consistent with a diagnosis of possible PNES.
– **Probable PNES**: cases in which the ictal events with a semiology indicative of PNES are witnessed by an expert clinician or assessed by video-EEG recording that also indicate no ictal epileptiform activity. Situations in which the observation of the onset of the ictal episode is missing or the evaluation is made by a clinician who lacks experience in ictal assessments make PNES “probable.”
– **Clinically established PNES**: cases in which an epilepsy specialist witnesses the episodes, and the semiotic and objective findings are compatible with PNES. That includes situations in which, for instance, there is resistance to the opening of the eyes, the interaction with the patient during the episode is possible as he/she maintains some level of consciousness and partial responsiveness, or the ictal episode ceases as the physician persuades the patient to terminate it. No epileptiform activity in interictal or ictal EEG can be found.
– **Documented PNES**: cases in which the diagnosis produced by an epilepsy specialist taking into account typical PNES semiology and no EEG-related epileptiform activity is found in any phase of the ictal event, or before and after it.

Clinicians have also tested the patient's responsiveness during PNES using different more or less invasive procedures. Old reports have indicated patients being pinched, stuck with a needle, splashed with water, or forced to inhale noxious chemical substances such as ammonia during or around a psychogenic attack in order to test the level of consciousness. However, there is no evidence that any of these invasive procedures are more effective than an intranasal tickle with a cotton swab (90).

## Supplementary Diagnostic Procedures

As mentioned above, only ictal EEG can be used to differentiate a subject suffering from PNES from a person affected by real epilepsy. However, many neurophysiologic, neuro-humoral, and neuropsychological tools can be used to identify at-risk subjects for PNES. These can help in conjunction with a thorough medical history, mental status, and neurologic examination.

### Blood Markers

Several serologic measures have been used to differentiate epilepsy from PNES. One of the most useful markers concerns the analysis of prolactin (PRL) levels (88, 91). Many studies have shown that the absence of postictal increases of PRL predicts PNES with an average sensitivity of 89% (92, 93). False-positive are usually due to the undergoing use of dopamine antagonists or tricyclic antidepressants as well as breast stimulation and the occurrence of syncope (93). PRL levels may also fail to rise after frontal lobe seizures. The American Academy of Neurology Therapeutics and Technology Assessment Subcommittee concluded that in samples collected 10–20 min after the onset of the ictal even, doubling of relative or absolute serum PRL levels (taking into account pre-ictal values) significantly helps to discriminate generalized tonic-clonic epilepsy from PNES (94). The analysis of serum levels of cortisol at baseline or after the dexamethasone suppression test does not reliably allow the differentiation between PNES, depression, or epilepsy (95). Increases in peripheral white blood counts, creatine kinase, and neuron-specific enolases have shown little discriminative power between PNES and true epilepsy (96). Compared to age-matched healthy controls, levels of the Brain-Derived Neurotrophic Factor (BDNF) are lower in patients with PNES but do not differ from patients with epilepsy (97).

### Neuroimaging Markers

As discussed above, structural imaging studies in patients with PNES have documented changes in the cortex and cerebellum (46). fMRI studies have also revealed changes in the functional connectivity that occurs between emotional, cognitive, and motor regions (42, 54). However, neuroimaging-related findings are of modest diagnostic value at the present time. It remains unclear whether these findings are specific to PNES, or can instead be tied to other comorbidities like depression, traumatic brain injury, etc., conditions that, it should be underlined, are frequently found in PNES patients. To date, only one fMRI study has examined the functional connectivity changes that are produced in PNES patients in response to external stimuli (54). The study suggested that PNES subjects exhibit a higher tendency to dissociate, a phenomenon that reflects the presence of hyperconnectivity between brain regions involved in emotion processing like the insula and motor regions adjacent to the precentral sulcus. The model is intriguing because it is the first to hypothesize a network-based mechanism for PNES. A recent investigation (98), using machine learning (ML), highlighted the role of selected cerebral areas that appear to be primarily involved in the clinical expression of PNES. The ML-based analysis revealed that the inferior frontal cortex (IFC), posterior cingulate cortex (PCC), and medial orbitofrontal cortex (OFC) are selectively activated in PNES patients. These findings are in line with the increased functional connectivity and reduced cortical thickness observed in these regions. OFC alterations have been consistently reported. It is conceivable that the altered communication between brain key regions involved in emotion regulation like the cingulate cortex, OFC, and frontal regions represents the neurobiological root of the dissociation process, by generating the disruption of information processing and aberrant sensorimotor activities. According to the author, these findings can be useful in distinguishing patients with PNES from controls at the individual level.

### Neuropsychological Tests

Neuropsychological tests can help to isolate distinct cognitive, emotional, and personality features of PNES patients, but have limited value for the differential diagnosis with epilepsy (99). PNES patients exhibit deficits in several cognitive domains (100). Many studies have examined the emotional factors associated with PNES and psychiatric comorbidity. PNES patients show a high presence of personality disorders. Studies aimed at differentiating PNES from epilepsy patients have made use of interview methods like the Structured Clinical Interview for DSM Diagnosis (SCID) or the Mini-International Neuropsychiatric Interview (MINI) as well as the Minnesota Multiphasic Personality Inventory (MMPI) or the MMPI-2. These studies have indicated that personality traits may differ when comparing patients with PNES top patients with PNES and epilepsy (100).

Personality traits, type of abuse, and age of onset of trauma vary as a function of the CD subtype. A recent study has shown that patients with PNES exhibited high scores in Neuroticism and low in Conscientiousness. Neuroticism-related features like anxiety, anger, hostility, depression, excessive self-consciousness, and vulnerability can be directly and specifically associated with the type of trauma reported by the patients. The Neuroticism domain describes a persistent, life-long tendency to experience life events negatively and has been associated with mood disorders (101). Neuroticism may represent a “distress proneness.” Thus, the higher neuroticism found in PNES patients indicates that they may be more sensitive to stressful events. Other studies have shown difficulties in coping with stress. Conscientiousness is frequently associated with higher levels of well-being and productivity, but can also predispose to experience more significant distress and difficulties in matching demanding tasks or situations. Conscientiousness has also been associated with self-oriented perfectionism (i.e., the tendency to set excessively high standards for oneself) as opposed to socially-oriented perfectionism (i.e., the tendency to believe that acceptance by others requires excessively high standard performances), a condition often associated with Neuroticism (102).

Research on the effects of traumatic experiences upon early childhood trauma indicates that severe early-life stress generates higher sensitivity of the hypothalamic-pituitary-adrenal axis in response to stressing situations that occur upon adulthood. Early traumatic experiences also produce greater vulnerability to depression (103). Moreover, PNES patients exhibit high rates of alexithymic personality traits (104). In a study focused on “psychosomatic” patients, Sifeos described a new personality trait which he, “for lack of a better term,” named alexithymic, a term derived from old from Greek that means “no words for mood” (105). Alexithymia is defined as the failure or difficulty in mentalizing, recognizing, and verbally describing emotional states, and is a well-documented risk factor for the development of depression (106). Thus, alexithymia is a relative constriction in emotional functioning, poverty of fantasy life, and inability to recognize and verbally describe one's emotions with appropriate words. The presence of alexithymia in PNES was investigated for the first time by Bewley and colleagues (107) who indicated higher levels of alexithymia in PNES patients when compared to epileptic patients. The developmental or biological etiology of alexithymia is still largely unknown. While some authors indicate that the disorder can develop as a maladaptive coping mechanism in response to trauma, only some scant neuroimaging-based evidence supports the notion that structural changes in the corpus callosum and frontal lobes are the anatomical substrate for alexithymia (108, 109).

## Epilepsy and PNES: The “Dual Diagnosis”

The PNES diagnosis is often complicated by the fact that epilepsy is a recognized risk factor for the development of PNES. About 10% of patients with PNES (68) also exhibit genuine epileptic seizures, a number likely higher when assessments are made by specialized centers. This condition is known as in epilepsy circles as “dual diagnosis.” Patients with “dual diagnosis” have similar demographic of PNES and epileptic patients (22). Mechanisms speculated to be at the basis of the development of PNES in epilepsy patient include (1) psychiatric comorbidities correlated to epilepsy, (2) the presence of a “seizure scaffold” on which PNES ensues; and (3) the development of substitute symptoms (in particular in patients recovering from epilepsy) to obtain secondary gains like caregiver attention, monetary compensation or work avoidance. Patients with pharmaco-resistant epilepsy are at higher risk of developing PNES, and vice versa. The dual diagnosis must be taken into account in cases of epilepsy patients showing unexpected patterns of seizures in terms of features and frequency. A dual diagnosis is harder than isolated PNES. No neurobiological or neuropsychological feature can be employed to differentiate these subjects from epileptic patients or PNES patients. Very few studies have attempted to assess the outcomes of these patients. Some data showed that the dual diagnosis predisposes to worse outcomes. However, once the correct diagnosis is made, the number of events and the use of ASD level off, thereby emphasizing the importance of a timely diagnosis of PNES in patients already affected by epilepsy.

## Pharmacoresistency and Pseudo-Refractory Epilepsy

The International League Against Epilepsy (ILAE) has identified PNES as one of the ten key neuropsychiatric conditions associated with epilepsy (1). Pharmacoresistency and pseudo-refractory epilepsy represent the other, critical, side of the PNES coin. One out of four to five patients admitted to video-EEG monitoring units with a diagnosis of pharmaco-resistant epilepsy is later found to suffer from non-epileptic events, the majority of which are of psychogenic origin (110, 111). The diagnostic differentiation between pseudo-refractory and true refractory epilepsy is essential to avoid unnecessary treatment, ASDs related side effects, and a negative effect on the quality of the patient life. Pharmaco-resistant epilepsy is defined as a neurological condition characterized by the failure to achieve a sustained seizure-free period in response to two courses of ASD (either as monotherapies or in combination) that are tolerated, appropriately chosen, and used with accurate titrations. The pseudo-intractability, instead, relates to the resistance to treatment that is caused by diagnostic errors. Pseudo-intractability is a condition relatively easy to manage but often underestimated and unrecognized in clinical practice. It should be stressed that not all patients with intractable epilepsy are truly pharmacoresistant (112, 113). Pseudo-intractability in epilepsy is still present, even at times in which sophisticated diagnostic and therapeutic options are available.

Future research will be needed to explore in more detail the clinical aspects as well as the psychopathological features of pseudorefractory epilepsy. The process will be helped by recruiting groups of subjects who exhibit selected types of psychopathology and different levels of trauma exposures.

## Management

The management of PNES is still largely unclear. A 2014 Cochrane review concluded that there is insufficient robust evidence to support any specific treatment for PNES (109). A 2017 study by Carlson and Perry (114) also indicated the absence of specific treatments and suggested the implementation of personalized approaches. Recent evidence suggests that psychological approaches may be the most effective way (115).

The management process should be divided into three stages. The initial stage concerns the communication of the diagnosis, a key step. Communication is facilitated by the presence of family members, a strategy that increases the understanding of the condition by patients and loved ones (70). The diagnosis must always be communicated with a tactful, empathic, and positive approach (70). The choice of the most appropriate words to be employed is a matter of a lively debate. Specific communication strategies have been published to maximize the efficacy of the process (38, 116). Terms like “hysterical seizures” and “pseudoseizures” are to be avoided and considered offensive. It is, however, questionable whether terms like “attack” (that can be associated with traumatic events sustained by the patient) or “seizure” (possibly generating confusion with real epileptic seizures) are more suitable (117). A small study of 13 PNES patients suggested that both terms were felt as problematic (118). A shared communication of the diagnosis increases the insight of the patient regarding his/her condition. Sometimes, communication of the PNES diagnosis can act as a therapeutic intervention. Recent studies have stressed out that most patients became PNES-free with time and after receiving a definite and clear diagnosis (119, 120).

The second stage of treatment involves acute therapeutic intervention. Detail psychiatric assessments should be arranged as psychiatric comorbidities are the rule and not the exception in PNES patients. Only 5% of patients do not exhibit the presence of comorbid psychiatric disorders or stressors (121). Predisposing, precipitating, and perpetuating factors must be investigated. Individualized psychotherapeutic and psychopharmacological treatment plans must be then set in place. PNES may be confused with panic attacks or associated with other CD like psychogenic movement disorders (122). Continued involvement of the epileptologist who has established the diagnosis is necessary to allow a safe tapering of ASD and treatment of any comorbid neurological condition. The treatment plan should include early tapering and discontinuation of ASD unless the patient showed specific beneficial effects by the use of ASD like, for instance, the antidepressant activity of lamotrigine or the mood-stabilizing effects of valproate. Sertraline or venlafaxine can be used to treat mood, anxiety, or psychotic disorders (70). There are no guidelines on the duration of any pharmacological and/or non-pharmacological treatment (123).

The final stage consists of the implementation of long-term interventions. The stage can make use of personalized interventions that include a long-term course of psychotherapy, case management as well as long-term pharmacological management of psychiatric comorbidity (70). Psychotherapy is considered the treatment of choice (124). Cognitive-behavioral therapy currently exhibits the most robust experimental and clinical evidence of efficacy (125–127). Individual or group psychodynamic therapy can also be considered (128–131). Psycho-educational approaches hold some promises (131). Unfortunately, compliance with psychotherapy and specifically CBT, of PNES patients is poor compared to patients affected by other psychiatric conditions (115). This reduced therapeutic compliance may be due to the scarcity of mental health services and mental health professionals, as well as the low confidence that patients exhibit toward this therapeutic approach (115).

## Conclusions

A better understanding of the complexity of PNES requires the concerted and coordinated efforts of neuroscientists, neurologists, psychiatrists, psychologists as well as, in our opinion, the creation of a multidisciplinary, multicultural/international study group set to develop a coherent research agenda and the promotion of large-size collaborative projects.

## Author Contributions

FA and FD contributed to the conception and design of the review. FA, FD, GE, MD, SS, and MO wrote the manuscript. All authors contributed to manuscript revisions, read and approved the submitted version.

## Conflict of Interest

The authors declare that the research was conducted in the absence of any commercial or financial relationships that could be construed as a potential conflict of interest.
